# Out-of-Hospital Cardiac Arrest and Airborne Fine Particulate Matter: A Case–Crossover Analysis of Emergency Medical Services Data in Indianapolis, Indiana

**DOI:** 10.1289/ehp.10757

**Published:** 2008-02-22

**Authors:** Frank. S. Rosenthal, John P. Carney, Michael L. Olinger

**Affiliations:** 1 School of Health Sciences, Purdue University, West Lafayette, Indiana, USA; 2 Department of Emergency Medicine, Indiana University School of Medicine, Indianapolis, Indiana, USA

**Keywords:** air pollution, cardiac arrest, cardiovascular system, case-crossover, EMS, environmental health, out-of-hospital, particulate matter

## Abstract

**Background:**

Previous studies have found particulate matter (PM) < 2.5 μm in aerodynamic diameter (PM_2.5_) associated with heart disease mortality. Although rapid effects of PM_2.5_ exposure on the cardiovascular system have been proposed, few studies have investigated the effect of short-term exposures on out-of-hospital cardiac arrest (OHCA).

**Objectives:**

We aimed to determine whether short-term PM_2.5_ exposures increased the risk of OHCA and whether risk depended on subject characteristics or presenting heart rhythm.

**Methods:**

A case–crossover analysis determined hazard ratios (HRs) for OHCAs logged by emergency medical systems (EMS) versus hourly and daily PM_2.5_ exposures at the time of the OHCA and for daily and hourly periods before it.

**Results:**

For all OHCAs (*n* = 1,374), exposures on the day of the arrest or 1–3 days before arrest had no significant effect on the incidence of OHCA. For cardiac arrests witnessed by bystanders (*n* = 511), OHCA risk significantly increased with PM_2.5_ exposure during the hour of the arrest (HR for a 10-μg/m^3^ increase in PM_2.5_ exposure = 1.12; 95% confidence interval, 1.01–1.25). For the subsets of subjects who were white, 60–75 years of age, or presented with asystole, OHCA risk significantly increased with PM_2.5_ during the hour of the arrest (HRs for a 10-μg/m^3^ increase in PM_2.5_ = 1.18, 1.25, or 1.22, respectively; *p* < 0.05). HR generally decreased as the time lag between PM_2.5_ exposure and OHCA increased.

**Conclusion:**

The results suggest an acute effect of short-term PM_2.5_ exposure in precipitating OHCAs, and a need to investigate further the role of subject factors in the effects of PM on the risk of OHCA.

In the last decade, several studies have associated exposure to airborne particulate matter (PM) with cardiac morbidity and mortality as well as effects on cardiac rhythms and electrocardiography. Although the exact nature of the mechanisms is still uncertain, some studies indicate that at least part of the increased mortality is attributable to acute cardiac events triggered by high PM levels. Attention has focused especially on the fraction of PM consisting of particles with aerodynamic diameter < 2.5 μm (PM_2.5_), which is considered a leading factor in inducing cardiovascular risks. For example, a hospital-based study by Peters et al. (2001) found that increased risk of myocardial infarction (MI) was associated with higher levels of PM_2.5_ in the 1- to 3-hr period immediately preceding the MI. Previous studies investigating a link between heart disease deaths and short-term increases in particulate air pollution have found mixed results. Levy et al. (2001) found no association between daily PM_2.5_ levels and out-of-hospital cardiac arrest (OHCA). Sullivan et al. (2003) found an association of OHCA with daily PM_2.5_ only for the subset of smokers with previously existing heart disease and only for exposure measured 2 days before the OHCA. Murakami and Ono (2006) found a significant increased risk of MI associated with 1-hr peaks of suspended PM.

Our goal was to investigate the effect of short-term exposure to PM_2.5_ on the incidence of OHCA, as reported in an emergency medical services (EMS) database. Additional objectives were to investigate the role of subject characteristics, that is, age, sex, race, and presenting heart rhythm on PM-induced risks; and to compare the effect of exposure averaging time and measurement method on the ability to detect an association between PM exposure and risk of OHCA. The study was conducted in Indianapolis, Indiana, a major metropolitan area with average annual PM_2.5_ levels close to the current U.S. Environmental Protection Agency (2007) standard of 15 μg/m^3^.

## Methods

### Study site and population

The Indiana University and the Purdue University Human Subjects Research Committees approved all data-collecting procedures. The Wishard Ambulance Service, which provides EMS to the population within the historic city limits of Indianapolis, provided data on OHCAs, collected according to Utstein guidelines (Cummins et al. 1991). The service makes approximately 65,000 emergency runs per year, of which approximately 1,000 per year are classified as cardiac arrest. Approximately 600 per year of the cardiac arrests result from trauma or are classified as dead on arrival (DOA; signs of rigor mortis, algor mortis, or livor mortis). Of the remaining 400 per year, approximately 150 per year are witnessed by bystanders. Data from 2 July 2002 to 7 July 2006 were used. We analyzed either all non-DOA incidents during the study period (*n* = 1,374) or all non-DOA incidents witnessed by bystanders (*n* = 511). We analyzed the witnessed cases in relation to hourly exposures because we assumed that the time of the cardiac arrest was known with greater accuracy compared with the other cases. The non-DOA cases, for which the time of incidence was considered less accurate, we analyzed in relation to daily average exposures. For each case, the following data were available: *a*) time of the initial call to EMS, *b*) time of arrival at site of the cardiac arrest, *c*) age, sex, and race of the subject, *d*) presenting heart rhythm (when available), *e*) ZIP code of the site location. We stratified some of the analyses by age, sex, and race in attempt to see whether particular subgroups were more susceptible to the effect of PM on OHCA. Analyses were also stratified by presenting heart rhythm in an attempt to investigate the characteristics of OHCAs associated with PM exposure.

### PM_2.5_ data

We obtained average daily PM_2.5_ values from data collected by the City of Indianapolis site 44 (Michigan Street) for 2002 and site 41 (Washington Park) for 2003–2006. These data were obtained according to the federal reference method (FRM) based on collection of 24-hr filter samples.

Hourly PM_2.5_ values were obtained from a tapered element oscillating microbalance (TEOM) monitor operated by the City of Indianapolis at site 41 at Washington Park. The validity of this measurement system can be affected by atmospheric conditions, particularly temperature, humidity, and precipitation. In extreme cases, these effects resulted in the monitor indicating negative values. At time points close to these negative values, readings were sometimes unusually high or low. Before the initiation of this study, the hourly data had been visually inspected by City of Indianapolis staff, and data points that appeared invalid either by negativity or lack of continuity with neighboring values were deleted. Approximately 2% of the hourly exposure values during the study period were missing in the database.

To correct for atmospheric effects on TEOM measurements, the hourly PM_2.5_ values were adjusted by two types of adjustments. From 1 January 2002 to 29 August 2003, TEOM measurements were used uncorrected for warmer months (April–October). For these months, daily TEOM 24-hr average values were in good agreement with data determined by the FRM. For colder months (November–March) TEOM readings were multiplied by the ratio of TEOM 24-hr averages to FRM measurements at the same or neighboring sites, averaged over all days from 2000 to 2003. From 29 August 2003 to 31 December 2006, TEOM readings were corrected by a two-segment linear model developed by Rizzo et al. (2003) that fits 24-hr averaged TEOM measurements with FRM measurements for the same days, incorporating the effect of function of ambient temperatures. Parameters in the Rizzo model were determined by fitting the model to FRM and TEOM data from site 41 for 2001 and 2004 (Childs A, personal communication). The overall validity of the TEOM data in our study is supported by a high correlation that we found between FRM values and average 24-hr TEOM values for the witnessed incident days in the study (*R*^2^ = 0.87, *p* < 0.0001).

**Figure 1:**
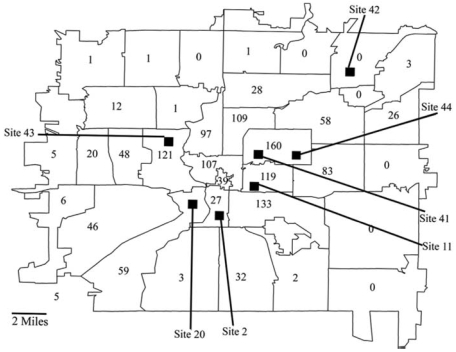
The population study area in Indianapolis, Indiana, with the number of non-DOA OHCAs in each ZIP code area and the location of the PM_2.5_ monitoring stations.

The two sites from which PM_2.5_ data were used were near the center of the population area in which the cardiac arrest cases occurred (Figure 1). Average daily data collected according to the FRM were available from several other monitoring sites every third day. We evaluated the degree to which the site 41 exposures were representative of the entire Indianapolis area by correlating the daily exposure values measured at site 41 with the daily exposure values measured at the other sites. The high correlations observed provided evidence that the exposures observed at site 41 were a suitable surrogate for ambient exposures in the entire study area (Table 1).

### Metereologic data

Hourly meteorologic data including temperature, relative humidity, and barometric pressure measured at the Indianapolis airport were obtained from the Midwest Regional Climate Center (Champaign, IL).

### Statistical analysis

We analyzed the data with a case–crossover study design (Levy et al. 2001; Neas et al. 1999) using conditional logistic regression, with the PM_2.5_ at the time of the OHCA being the exposure of the case. Referent exposures, selected by time-stratified sampling, were the exposures on all days falling within the same month and on the same day of the week as the case. Hazard ratios (HRs) expressing the increased risk for an increase of 10 μg/m^3^ in PM_2.5_ exposure were computed using PROC LOGISTIC under SAS version 9 (SAS Institute Inc., Cary, NC). A subsample of analyses calculated with PROC LOGISTIC, using the exact computational option, yielded identical results to those done with the standard procedure. In the analyses, HRs were adjusted for temperature, relative humidity, and barometric pressure, by including these factors in the statistical model.

For analyses of the witnessed non-DOA OHCAs, the exposure of the case was taken as either the exposure for the hour in which the OHCA occurred or the exposure during an hour a specific number of hours before the OHCA occurrence (lagged exposures). The exposure during the hour in which the OHCA occurred was designated lag0. The exposure during the hour preceding the OHCA was designated lag1. The exposure during the 1-hr period beginning 2 hr before the OHCA was designated lag2, and so on. Additional analyses were done for the 4-hr average of lag0, lag1, lag2, and lag3 (designated lag03), the 8-hr average of lag0, lag1, lag2, lag3, lag4, lag5, lag6, and lag7 (designated (lag07), and the 24-hr average of lag0, lag1…, lag23 (designated lag023). For the average exposures (lag03, lag07, lag023), values were considered missing if < 75% of the hours needed for the average were available. Meteorologic variables were used with the same lag period as the particle exposures. For analysis of a given lagged exposure, a case was dropped if exposure data and meteorologic data were not available for the case and at least one referent. In the hourly analyses, the number of dropped cases varied with lag number and ranged from 16 to 21 (of 511).

For analysis of all non-DOA cases, we used daily (FRM) exposure data. The exposure of the case was taken as either the exposure for the day in which the OHCA occurred or the exposure for a day a specific number of days before the OHCA occurred (lagged exposures). The exposure for the day in which the OHCA occurred was designated lag0d. The exposure for the preceding day was designated lag1d. The exposure for the day 2 days before the day of the case was designated as lag2d, and so on. Additional analyses were done for the 2-day average of lag0d, lag1d (designated lag01d), the 3-day average of lag0d, lag1d, lag2d (designated lag02d), and the 4-day average of lag0d, lag1d, lag2d, and lag3d (designated lag03d). Additional analyses were done for the average of lag0d and lag1d (designated lag01d), the average of lag0d, lag1d, and lag2d (designated lag02d) and the average of lag0d, lag1d, lag2d, and lag3d (designated lag03d). For lag01d, lag02d, lag03d, values were considered missing if any of the values needed for computing them were not available. Daily average values of temperature, relative humidity, and barometric pressure were used in these analyses and were used with the same lag period as the corresponding particulate values. HRs for the daily exposures were computed with statistical procedures similar to those computed for the hourly exposures. For analysis of a given lagged daily exposure, a case was dropped if exposure and meteorologic data were not available for the case and at least one referent day. In the analysis of non-DOA cases, the number of dropped cases varied with daily lag and ranged from 26 to 31 (of 1,374).

## Results

### Population and exposure characteristics

The demographic characteristics of the cases varied slightly depending on whether cases were witnessed or unwitnessed and on the presenting cardiac rhythm (Tables 2 and 3). Percentiles of daily exposures (determined according to the FRM) for the non-DOA and of hourly exposures for the non-DOA witnessed cases are shown in Table 4.

### Analysis of non-DOA cardiac arrests

**Figure 2:**
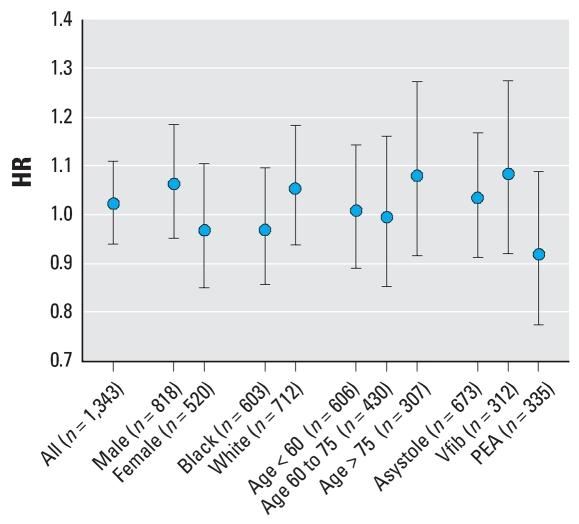
HRs for a 10-μg/m^3^ increase in daily PM_2.5_ (FRM) exposure for non-DOA OHCA. Abbreviations: PEA, pulseless electrical activity; Vfib, ventricular fibrillation. Exposure is determined for the day on which the cardiac arrest occurred (lag0d). Error bars indicate 95% confidence intervals.

For all (witnessed and unwitnessed) non-DOA cases, no statistically significant associations between OHCA and PM_2.5_ daily exposure were found. Similarly, there was no significant effect for any stratum when cases were stratified by race, sex, age group, and presenting cardiac rhythm (Figure 2). Similarly, there was no significant association between OHCA risk and PM_2.5_ daily exposure, for all subjects and for subjects stratified by heart rhythm, for any of the lagged exposures (Table 5).

### Analysis of witnessed non-DOA cardiac arrests

**Figure 3:**
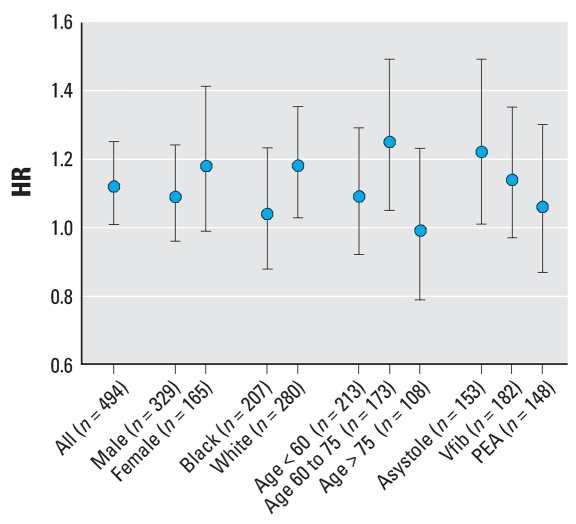
HRs for witnessed non-DOA OHCAs for a 10-μg/m^3^ increase in PM_2.5_ exposure during the hour of the arrest. Abbreviations: PEA, pulseless electrical activity; Vfib, ventricular fibrillation. Results are shown for all subjects and for subjects grouped by sex, race, age, and presenting heart rhythm. Error bars indicate 95% CIs.

**Figure 4:**
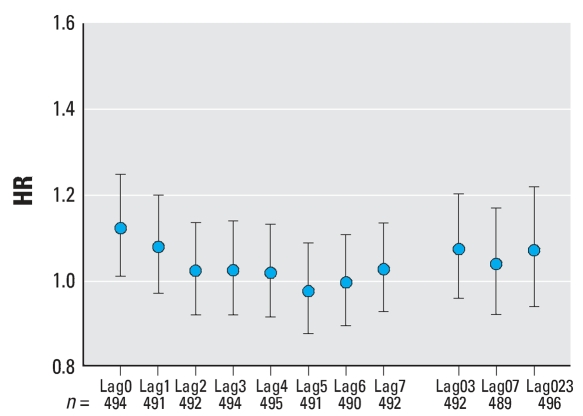
HRs for witnessed non-DOA OHCAs by lagged PM_2.5_ exposure. The HR shown is for a 10-μg/m^3^ increase in PM_2.5_ exposure. Lag0 indicates exposure determined during the hour of the cardiac arrest, lag1 indicates exposure determined 1 hr before the cardiac arrest, etc. Lag03 is the average exposure for the hour of the cardiac arrest and 3 hr preceding. Lag07 is the average exposure for the hour of the cardiac arrest and the 7 hr preceding. Lag023 is the average exposure for the hour of the cardiac arrest and the 23 hr preceding. Error bars indicate 95% CIs.

**Figure 5:**
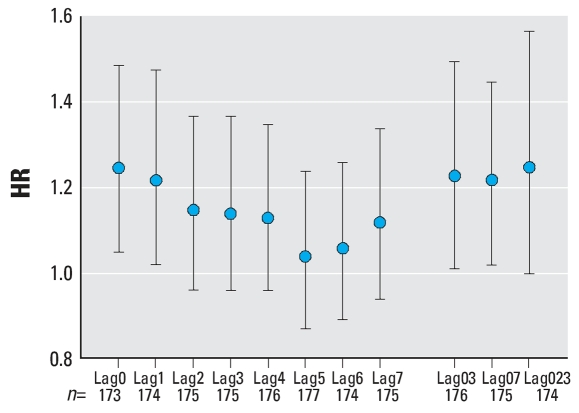
HRs for witnessed non-DOA OHCAs, for subjects aged 60–75 years, by lagged PM_2.5_ exposure. The HR shown is for a 10-μg/m^3^ increase in PM_2.5_. For explanation of lag0, etc., see Figure 4. Error bars indicate 95% CIs.

**Figure 6:**
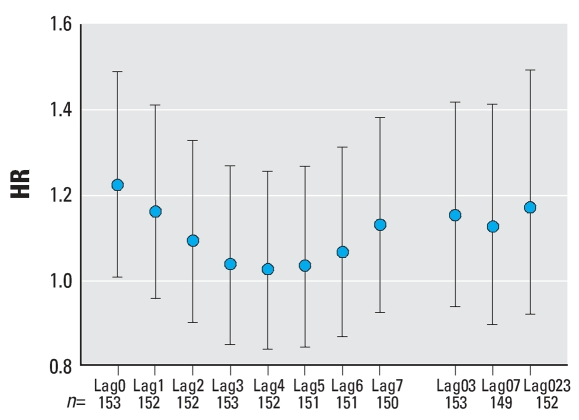
HRs for witnessed non-DOA OHCAs, for subjects presenting with asystole, by lagged PM_2.5_ exposure. The HR shown is for a 10-μg/m^3^ increase in PM_2.5_. For explanation of lag0, etc., see Figure 4. Error bars indicate 95% CIs.

Analysis of all non-DOA witnessed cardiac arrests found a statistically significant HR of 1.12 [95% confidence interval (CI), 1.01–1.25) for lag0. When analyzed for subject characteristics and for lag0, there were significant HRs for white subjects (HR = 1.18; 95% CI, 1.03–1.35), for subjects in the group 60–75 years of age (HR = 1.25; 95% CI, 1.05–1.49), and for subjects presenting with asystole (HR = 1.22; 95% CI, 1.01–1.59) (Figures 3–6). For subjects 60–75 years of age, the HR was also significantly > 1 for lag1, lag03, and lag023 (*p* < 0.05). Although generally > 1, no other HRs among subgroups of subjects by age, sex, race, or presenting heart rhythm were statistically significant for any lag. None of the differences between HRs by race, sex, age group, or presenting heart rhythm were statistically significant (*p* > 0.05). In general, the HR decreased with lag from lag0 to lag5 (Figures 4–6).

## Discussion and Conclusions

The principal findings of this study are marginally statistically significant associations of hourly PM_2.5_ exposure with OHCAs witnessed by bystanders, for all subjects, for white subjects, for subjects 60–75 years of age, and for subjects presenting with asystole (*p* < 0.05).

Although the differences we found in HR according to race, age, and presenting heart rhythm may be attributable to chance, they may also indicate varied susceptibilities to the effects of PM. Subjects presenting with asystole were slightly older and slightly more commonly female compared with other subjects in our study (Table 3), factors that have been associated with both presentation of asystole at cardiac arrest and sensitivity to PM-induced health effects. (Chen et al. 2005; Gold et al. 2000; Liao et al. 1999; Pope et al. 1999; Wigginton et al. 2002). It is possible that those presenting with asystole included a number of subjects whose cardiopulmonary arrest was secondary to respiratory failure during an acute deterioration of asthma or chronic obstructive pulmonary disease (COPD), brought about by particulate exposure. Herlitz et al. (1996) found that OHCAs caused by the deterioration of COPD were much more likely to present with asystole (50%) than with ventricular fibrillation (7%). Further studies detailing the medical histories of the subjects might shed light on the role of COPD and other preexisting disease in PM-related OHCA. Both the elevated HR for white subjects and those in the age bracket 60–75 years were unexpected in that they are opposite to the general patterns of cardiac arrest risk versus age and race that have previously been noted (Becker et al. 1993; Iwami et al. 2003). However, a previous study of mortality versus daily exposure to PM with diameter < 10 μm aerodynamic diameter (PM_10_) found a statistically significant excess mortality risk due to MI, associated with PM_10_ exposure, in a middle-age bracket (65–75 years), whereas the excess risks in both younger and older age brackets were several times smaller and not statistically significant (Zeka et al. 2006).

Many studies have identified relationships between heart disease mortality and particulate air pollution. However, causative mechanisms are still unclear, as is the relationship of acute and chronic effects. Two major acute effects of particulate exposure on the cardiovascular system have been proposed: *a*) Particulate exposure may cause inflammation and an increase in blood coagulability leading to increased risk of coronary blockages; and *b*) particulate exposure may act on the autonomic nervous system to cause increase vulnerability to heart arrhythmias. Two studies have found chemical markers associated with blood coagulability increased in particulate-exposed subjects. (Baccarelli et al. 2007; Pekkanen et al. 2000). An additional study by Pekkanen et al. (2002) found that in subjects with coronary heart disease, exposure to particulates was associated with an increased incidence of ST-segment depression during exercise, a marker of myocardial ischemia. Several studies have found decreases in heart rate variability (HRV; a marker of cardiac autonomic control) associated with particulate exposure (Gold et al. 2000; Liao et al. 1999; Magari et al. 2002; Pope et al. 1999).

In this study, the finding of a relationship between exposure and cardiac arrest using hourly exposure data but not with daily exposure is consistent with a rapid effect of particulate air pollution on the cardiovascular system. Such a rapid response is supported by the fact that the highest HR occurred for lag0 versus later lags, and the fact that there was general downward trend of HR with lag number (Figures 4–6). Previous studies also support the hypothesis of a rapid-acting effect of air pollution on the cardiovascular system. For example, Peters et al. (2001) studied 772 MI patients and found a significant association of the time of occurrence of the MI with PM_2.5_ for lag0 and lag1 but not for other lags. Furthermore, studies of HRV versus particulate exposure have found decreased HRV associated with PM_2.5_ exposure in the period of 1–4 hr before the HRV measurements were made (Magari et al. 2001, 2002).

Our current study may be compared with a study by Levy et al. (2001) of OHCA versus PM_10_ levels in Seattle, Washington, a city with PM levels similar to those in Indianapolis. As in the present study, these authors found no effect of daily PM exposure on OHCA incidence. However, the ability of the study by Levy et al. (2001) to detect any PM–OCHA relationship may have also been limited by the fact that the study excluded OHCAs associated with a history of heart disease or life-threatening conditions including end-stage lung, liver, or renal disease.

Although some studies point to an acute effect of particulate exposure on cardiac disease, the possibilities of short-term and longer-term effects of PM on the cardiovascular system are not mutually exclusive. In fact, three time scales of action after peak exposures can be considered, one on the level of minutes and hours, one on a level of days, and one for longer time periods. Indeed, in the study by Peters et al. (2001), a multiple regression analysis found that both the 2-hr average exposure and the 24-hr average exposure contributed to the risk of MI. In the study by Pekkanen et al. (2002) identifying ischemia during exercise, the HRs were larger for a lag of 2 days than for lags of 0 or 1 day.

Assuming a strong short-term component of cardiac responses to air pollution, the precision of relating the time of cardiac arrest to measurement of exposure may be critical in detecting an exposure–response relationship. In this study, the use of daily FRM values as a measure of exposure can be considered an estimate of an average exposure over many hours before the event or a surrogate estimate of the exposure at the time of the event. This surrogate measure is imprecise because of the variation of PM_2.5_ values throughout the day, and if the incident time is early in the day, the average may include many PM_2.5_ values at time points after the incident occurs.

The magnitude of the HRs seen for all witnessed OHCAs and for those witnessed OHCAs presenting with asystole may be compared with previous studies that found associations between hourly PM exposures and heart disease or heart disease mortality. In their study of the occurrence of MI, Peters et al. (2001) found an HR of 1.48 for a 25-μg/m^3^ increase in the 2-hr previous PM_2.5_ exposure, and 1.69 for a 25-μg/m^3^ increase in the 24-hr PM_2.5_ exposure. For comparison, the HR computed for a 25-μg/m^3^ increase in the current study for OHCAs witnessed by bystanders is 1.33. A recent study in Tokyo by Murakami and Ono (2006) found rate ratios of 1.13–1.18 when the incidence of MIs in 1-hr windows after PM_2.5_ exposures > 100–249 μg/m^3^ was compared with the incidence of MIs in 1-hr windows after PM_2.5_ exposures < 100 μg/m^3^. Sullivan et al. (2005) studied particulate air pollution levels, measured by nephelometry, at 1, 2, and 4 hr before MI onset and found a close to significant association of MI onset with the pollution level 1 hr before onset (HR for a 10-μg/m^3^ increase in PM = 1.01; 95% CI, 0.98–1.05). Other studies have reported increased risks associated with daily measurements of PM. For example, Sullivan et al. (2003) found significant associations of primary cardiac arrest with PM_2.5_ exposure in current smokers with preexisting heart disease for exposure measured 2 days before the arrest (HR for a 13.8 μg/m^3^ increase in PM_2.5_ = 1.06; 95% CI, 1.06–1.55). The variety of risk estimates in the various studies may reflect differing time frames of exposure measurement, different compositions of PM, different characteristics of the exposed populations, and considerable statistical uncertainties.

### Limitations of the current study

A principal limitation of the study was the small number of cases available. This made it difficult to determine, with precision, effects (or lack of effects) as well as to distinguish the effects of race, age, sex, and presenting heart rhythm. The number of subjects was limited by size of the study population and the number of years available for study in which both EMS and hourly PM_2.5_ data were available. Nevertheless, it is striking that in small numbers of subjects (e.g., approximately 170 for witnessed non-DOA subjects, 60–75 years of age), we were apparently able to discern an effect of PM on OHCA. Studies in larger metropolitan areas with similar levels of air pollution may provide further insights into the risks and the factors associated with them. This study underscores the importance of obtaining valid historic PM_2.5_ hourly measurements in large urban areas.

An additional key limitation of this study is that the database does not provide sufficient information to determine the etiology of the cardiopulmonary arrests. The study population includes all patients for whom a 911 system responded and who were in cardiopulmonary arrest on the arrival of the EMS crews and were not pronounced DOA by the crews. These deaths may have had many causes including respiratory failure after deterioration of asthma and COPD, pulmonary emboli, drug overdoses, ruptured abdominal aortic aneurysms, or aortic dissections, in addition to acute coronary syndromes.

The imprecision of exposure estimates also limited this study. Ideally, one would like to know the exact exposure of an individual in the hours immediately preceding cardiac arrest. We were limited to an ambient PM_2.5_ reading of a monitor based in central Indianapolis. Although correlation analysis provides evidence that PM_2.5_ levels were approximately uniform over the study area (Table 1), there may be some variation between ambient level at the monitor and where the cardiac arrest occurred. Furthermore, the cardiac arrest may have occurred indoors, where PM_2.5_ are different from ambient levels. However, this study is similar to other studies that have been able to detect cardiovascular effects using outdoor ambient levels. The ability of these studies to detect cardiovascular effects of PM using outdoor ambient levels may be related to a moderately strong correlation between indoor levels and outdoor ambient levels and the hypothesis that the fraction of indoor PM_2.5_ arising from outdoor sources is the most toxic in terms of cardiovascular effects. In a study of 58 homes of nonsmokers, Leaderer et al. (1999) found that approximately 75% of PM_2.5_ during summer months was associated with outdoor sources. Janssen et al. (2005), studying cardiovascular patients in Amsterdam and Helsinki, found strong correlations between indoor, outdoor, and personal levels of PM_2.5_. Analyzing the particulates by composition, they found the strongest correlations for sulfur and particle absorbance (an indicator of vehicular traffic pollution), both primarily associated with outdoor sources (median Spearman *r* > 0.9).

To the extent that the imprecision of exposure estimates in this study can be considered nondifferential with respect to OHCA risk, the HRs in this study may be considered to underestimate the true HRs in the study population.

Another limitation of this study is that co-pollutants were not assessed. Previous studies have associated both ultrafine particulates and ozone with heart disease mortality. For example, a study by Forastiere et al. (2005), using a case–crossover approach to study out-of-hospital coronary death, found a 7.6% increase in mortality associated with an interquartile difference of particle number concentration (a measure of ultrafine particulate). To the extent that the levels of these co-pollutants are correlated with the PM_2.5_ values measured in this study, these pollutants, rather than or in addition to PM_2.5_, may have contributed to the HRs measured here. Further studies can examine this possibility with multivariate models including assessment of various pollutants simultaneously.

A final limitation of the study was the lack of information on various factors that may have increased the risk of cardiovascular disease in the subjects (e.g., obesity, fitness, stress). Future studies examining these factors may be able to identify population subsets that are especially sensitive to the cardiovascular effects of particulate exposure.

## Figures and Tables

**Table 1:** Correlation of daily PM_2.5_ values at Indianapolis sites with daily PM_2.5_ values measured at site 41 for 2002–2006.

Site	*R*^2^	Slope	*p*-Value
2	0.94	0.98	< 0.0001
11	0.91	0.91	< 0.0001
19	0.92	1.01	< 0.0001
42	0.94	1.03	< 0.0001
43	0.96	0.93	< 0.0001
44	0.97	0.96	< 0.0001

**Table 2:** Characteristics of non-DOA OHCAs.

	All	Witnessed	Unwitnessed
No.	1,374	511	855
Age (mean ± SD)	60.4 ± 18.9	60.9 ± 17.4	60.0 ± 19.7
Race (%)			
White	53.3	56.8	51.7
Black	44.7	41.9	46.6
Other	1.7	1.4	1.8
Sex (%)			
Male	60.6	66.5	57.4
Female	39.0	33.5	42.6
Presenting rhythm (%)			
Asystole	50.2	30.9	62.0
Vfib	23.2	37.2	15.0
Vtach	0.7	1.8	0.1
PEA	25.0	29.8	22.2
Unknown	1.0	0.4	0.7

Abbreviations: PEA, pulseless electrical activity; Vfib, ventricular fibrillation; Vtach, ventricular tachycardia.

**Table 3:** Characteristics of non-DOA OHCAs witnessed by bystanders.

	Presenting heart rhythm				
	All	Vfib	Asystole	PEA	Vtach
No.^a^	511	190	158	152	9
Response time^b^	4.17 ± 2.39	4.19 ± 1.57	4.01 ± 1.69	4.09 ± 1.70	3.94 ± 2.09
Age (years)					
Mean ± SD	60.9 ± 17.6	9.0 ± 16.2	62.3 ± 20.0	61.7 ± 16.7	61.4 ± 16.9
% < 60	43.1	47.4	38.6	42.8	22.2
% 60–75	34.8	37.9	29.8	34.9	55.6
% > 75	22.1	14.2	31.7	22.4	22.2
Race (%)					
Black	41.9	40.0	41.8	44.7	44.4
White	56.8	58.4	57.0	54.6	44.4
Sex (%)					
Male	66.5	71.1	60.8	66.4	66.7
Female	33.5	29.0	39.2	33.6	33.3

Abbreviations: PEA, pulseless electrical activity; Vfib, ventricular fibrillation; Vtach, ventricular tachycardia.

a Two of the 511 cases have unknown rhythms.

b Arrival time – dispatch time [min (mean ± SD)].

**Table 4:** Distribution of PM_2.5_ levels for index and referent days and hours.

		Percentile				
	No.	10th	25th	50th	75th	90th
All non-DOA (daily FRM values)						
All heart rhythms	3,882	6.4	9.4	13.9	19.5	25.8
OHCAs	1,294	6.7	9.6	14.1	19.5	25.8
Referents	2,588	6.3	9.3	13.9	19.5	25.8
Asystole	1,962	6.4	9.2	13.8	19.4	24.8
OHCAs	654	6.3	9.2	13.9	19.7	25.8
Referents	1,308	6.4	9.2	13.8	19.2	24.3
Witnessed non-DOA hourly (hourly TEOM values)						
All heart rhythms	1,416	5.6	8.8	13.8	20.7	29.8
OHCAs	472	5.3	8.8	13.6	21.9	30.5
Referents	944	5.7	8.8	13.9	20.4	29.0
Asystole	438	5.8	8.5	13.2	19.8	27.9
OHCAs	146	6.2	9.4	14.7	21.3	30.5
Referents	292	5.6	8.3	12.7	19.1	27.0

**Table 5:** HRs for out-of-hospital non-DOA cardiac arrests versus daily FRM exposures (measured according to the FRM) by presenting heart rhythm and daily lag.

	All		Asystole		Vfib		PEA	
Lag^a^	No.	HR (95% CI)	No.	HR (95% CI)	No.	HR (95% CI)	No.	HR (95% CI)
Lag0d	1,343	1.02 (0.94–1.11)	673	1.03 (0.91–1.17)	312	1.08 (0.92–1.28)	335	0.92 (0.77–1.08)
Lag1d	1,340	1.00 (0.92–1.08)	675	1.00 (0.89–1.13)	309	1.02 (0.87–1.21)	333	0.98 (0.83–1.15)
Lag2d	1,346	0.98 (0.90–1.06)	677	1.01 (0.90–1.13)	312	0.96 (0.80–1.14)	335	0.96 (0.82–1.14)
Lag3d	1,348	1.00 (0.92–1.08)	673	0.98 (0.87–1.10)	314	1.10 (0.93–1.31)	338	0.95 (0.82–1.10)
Lag01d	1,323	1.02 (0.92–1.12)	665	1.03 (0.90–1.18)	308	1.06 (0.88–1.28)	327	0.96 (0.80–1.17)
Lag02d	1,302	1.01 (0.91–1.12)	655	1.05 (0.90–1.22)	304	1.01 (0.82–1.25)	321	0.98 (0.80–1.21)
Lag03d	1,288	1.02 (0.91–1.14)	644	1.04 (0.88–1.22)	303	1.05 (0.83–1.32)	319	0.98 (0.78–1.21)

Abbreviations: PEA, pulseless electrical activity; Vfib, ventricular fibrillation.

a Lag0d, exposure on day of cardiac arrest; Lag1d, exposure on day before cardiac arrest, etc.; lag01d, average of lag0d and lag1d; lag02d, average of lag0d, lag1d, and lag2d; lag03d, exposure of lag0d, lag1d, lag2d, and lag3d.
